# Supplementary material for: Technical pipeline for screening microbial communities as a function of substrate specificity through fluorescent labelling
Source: Commun Biol. 2022 May 11;5:444. doi: 10.1038/s42003-022-03383-z (PMC9095699; doi:10.1038/s42003-022-03383-z)
Supplement: Supplementary file 2 — Supplemental Information [file 42003_2022_3383_MOESM2_ESM.pdf]

Preparation of 2-AB labelled standards - X 1-6 & M 1-6 (HPLC-HILIC-FLD chromatograms & MS)

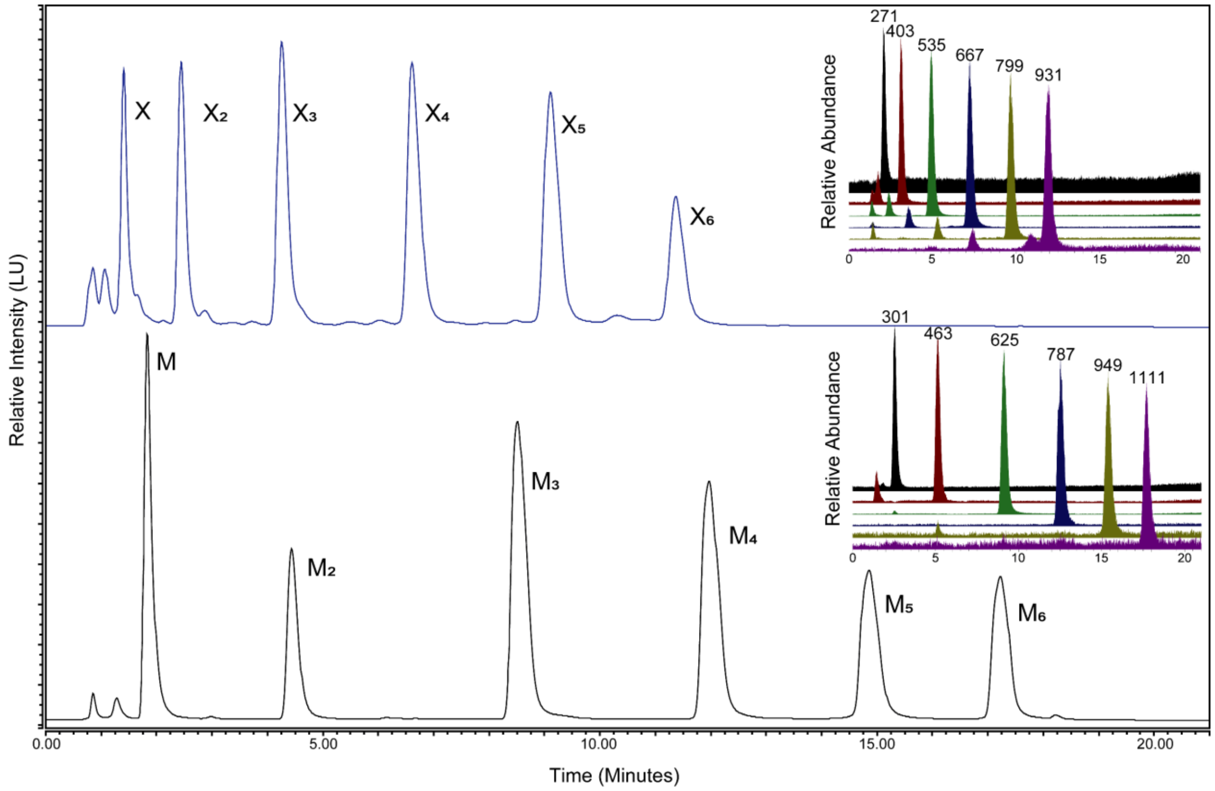

Supplementary Figure 1 – HPLC-HILIC-FLD chromatograms of 2-AB labelled DP 1-6 xylose and mannose. *Inset* Accompanying, confirmatory mass spectrometry profile peaks with *m/z* values of each labelled compound – all masses are protonated products. Abbreviations: X, xylose; X_2, xylobiose, X_3, xylotriose, X_4, xylotetraose, X_5, xylopentaose, X_6, xylohexaose; M, mannose; M_2, mannobiose, M_3, mannotriose, M_4, mannotetraose, M_5, mannopentaose, M_6, mannohexaose.

Supplementary Note 1: Substrate purification and excess label removal (HPLC-HILIC-FLD chromatograms and MALDI-ToF)

A number of solvents including chloroform, octanal and ethyl acetate were investigated for their effectiveness in removing excess free label in solution after the labelling synthesis. While octanal extraction proved highly efficient - 90+% free 2-AB removed when monitored by HPLC-FLD. However, on closer inspection by mass spectrometry methods we also noted a major issue with this technique. When substrates were characterised further using FLD-MS and MALDI-ToF it was determined that octanal used in the extraction of free 2-AB, was found to actually have been bound to the labelled carbohydrates in solution, an issue which had been previously observed[1]. Both chloroform and ethyl acetate, while providing a slightly lower efficiency in removing excess label than octanal were both deemed suitable for use going forwards. Ethyl acetate was ultimately chosen due to the toxicity and handling issues associated with chloroform usage.

As commonly reported solid phase extraction (SPE) can be effectively used to purify labelled glycans, however, when testing a couple of such examples (Supelco DSC-18 & ENVI CARB) with our substrates we observed appreciable losses and as such were not used going forwards.

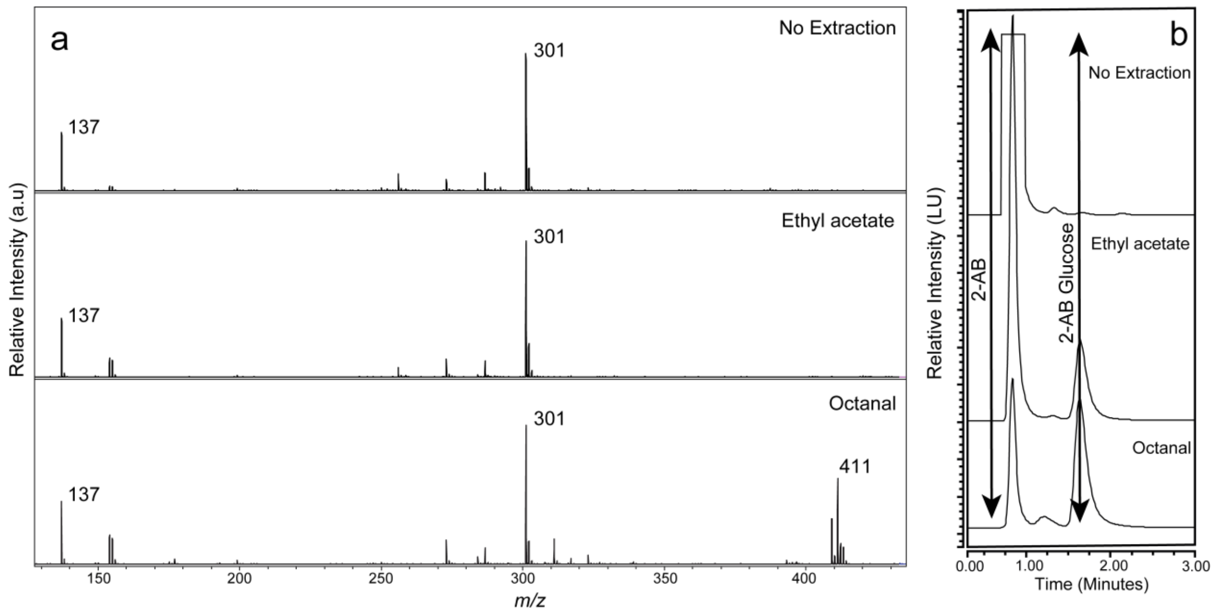

Supplementary Figure 2 – Analysis of substrate purification determination by MALDI-ToF and HPLC-HILIC-FLD. **a** – MALDI-ToF profile of 2-AB labelled glucose (*m/z* 301) demonstrating the extra peak at *m/z* 411 present in the octanal extracted sample. *Top* – No extraction, *Middle* – Ethyl acetate extraction, *Bottom* – Octanal extraction. Excess 2-AB is observed as *m/z* 137. All *m/z* appear as protonated adducts **b** - Accompanying HPLC-HILIC-FLD chromatograms demonstrating varying degrees of excess 2-AB removal – *Top* – No extraction, *Middle* – Ethyl acetate extraction, *Bottom* – Octanal extraction.

Demonstration of scale up capabilities (HPLC-HILIC-FLD chromatograms)

Following initial labelling experiments, generally carried out on 1-100 mg scale to optimise reaction conditions, the process was successfully scaled up. Labelled products were generated in different amounts (up to 12 g) and compared using the established analytical methodologies.

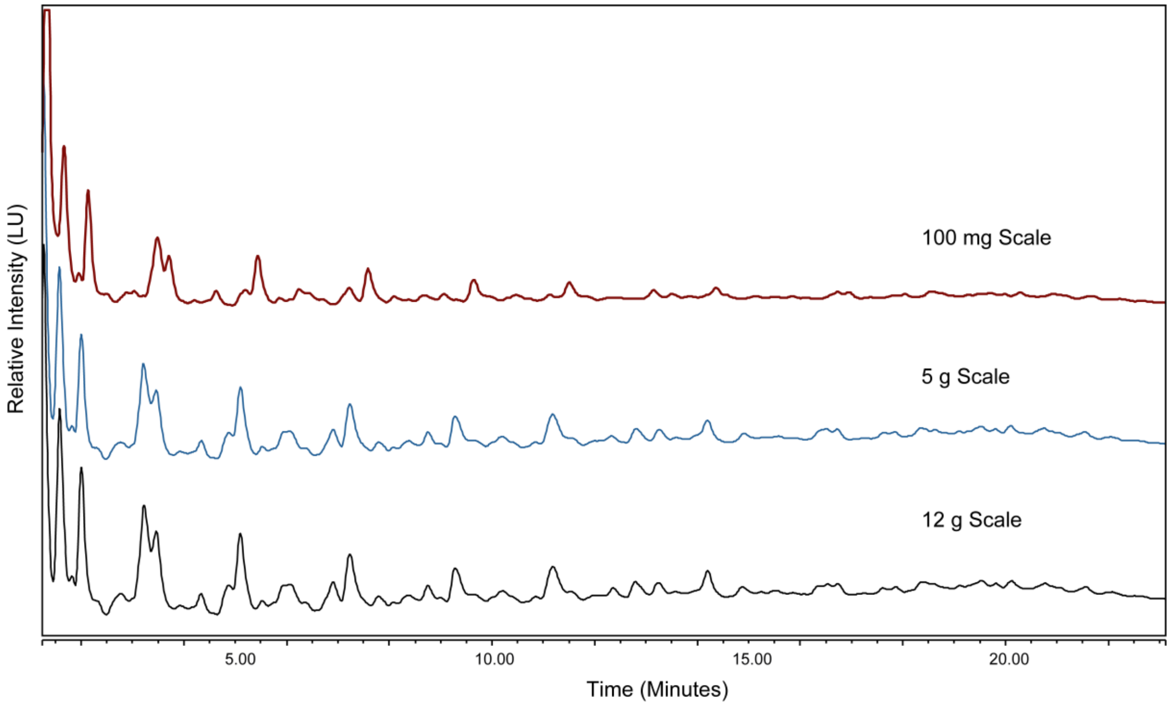

Supplementary Figure 3 – HPLC-HILIC-FLD chromatograms of 2-AB labelled GH26-AcGGM performed at different scales. The retention of fine detail, arising from a complex mix of acetylated oligosaccharides is retained throughout the scaling of the labelling procedure – 100 mg (red), 5 g (blue) and 12 g (black).

Additional analytical data for screening co-culturing experiments (MALDI-ToF and HPLC-HILIC-FLD)

Despite observing relatively low inclusion levels from the flow cytometry experiments for all of the fermentations conducted with the shorter and less complex labelled substrates, GH10-AGX and GH26-AcGGM, analytical observations showed some differences. An example of such a deviation is given in Supplementary Figure 4 below.

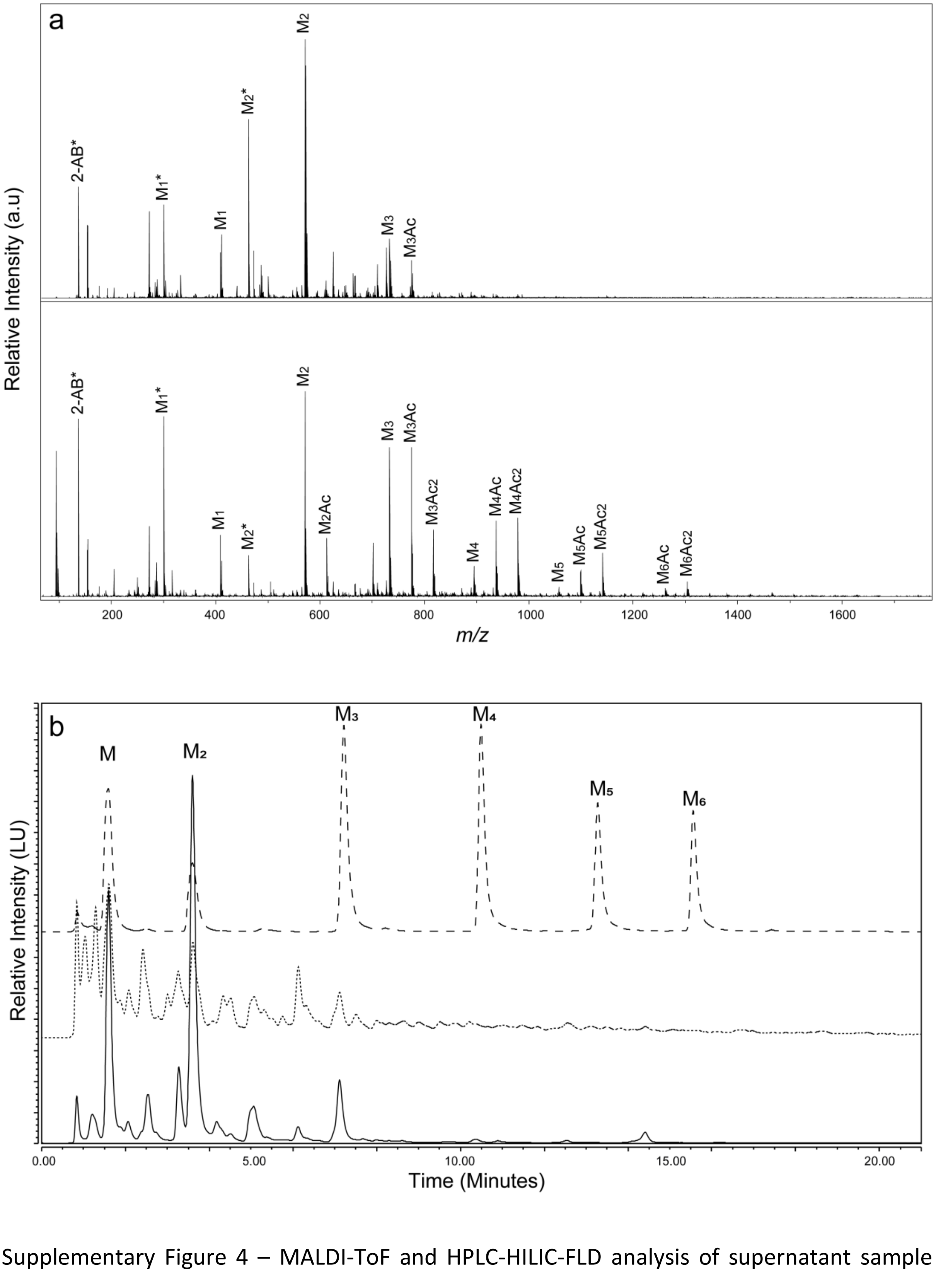

Supplementary Figure 4 – MALDI-ToF and HPLC-HILIC-FLD analysis of supernatant sample from *B. ovatus + B. cellulosilyticus* fermentation with 2-AB labelled GH26-AcGGM. **a** – MALDI-ToF of *Top* – 72 hour supernatant sample from *B. ovatus + B. cellulosilyticus* fermentation

with 2-AB labelled GH26-AcGGM and *Bottom* Starting substrate of 2-AB labelled GH26-AcGGM – all peaks appear as octanal conjugated products (*m/z* = 108/110) unless designated by * which indicates protonated. Peaks corresponding to Mannose (*m/z* 301* & 411) and Mannobiose (*m/z* 463* & 571) are by far the most prevalent structures observed in the supernatant. **b** - HPLC-HILIC-FLD chromatograms of Man 1-6 standards (dashed line), 2-AB labelled GH26-AcGGM (dotted line) and 72 hour supernatant sample from *B. ovatus + B. cellulosilyticus* fermentation with 2-AB labelled GH26-AcGGM (Solid line). Dominant peaks corresponding to mannose and mannobiose are observed in the fluorescence chromatogram from 72 hours, indicating a build-up and an overall non-degradability of these labelled glycans. Abbreviations: M, mannose; M_2, mannobiose, M_3, mannotriose, M_4, mannotetraose, M_5, mannopentaose, M_6, mannohexaose; Ac, acetyl group.

Additional co-culturing results (Flow data and HPLC-HILIC-FLD chromatograms)

AcAGX was chosen as a model substrate to further study the labelling process. A number of strains of *Bacteroides* were analysed to study uptake of labelled glycans over time. *B. cellulosilyticus* as a single culture demonstrated considerable labelled substrate incorporation after 24 hours, whilst far lower uptake (confirmed by FACS) was observed in all other co-cultured fermentations after the same time period. After 72 hours it could be observed that *B. cellulosilyticus* on its own once again showed more incorporation of the labelled material. However, it should be noted that all other co-cultured strains demonstrated a marked increase in uptake at this time point (Supplementary Figure 5). There are several possible explanations for this, three of which are i) *B. cellulosilyticus* in combination with other strains causes a delay to label uptake; ii) adaptation of the bacteria to utilize the substrate over the time frame of the experiment, hence an extended lag phase due to suboptimal growth conditions; iii) cross-feeding of the different strains – *B. cellulosilyticus* producing more readily accessible carbohydrates for other organisms, which has been shown recently to be a viable possibility[2]. Whilst we also recognise that the small amount of growth after 72 hours in the *B. caccae* + *B. theta* is unexpected, it may be due to some shorter oligosaccharides present that can be taken up via other uptake channels other than the specific xylan transporter.

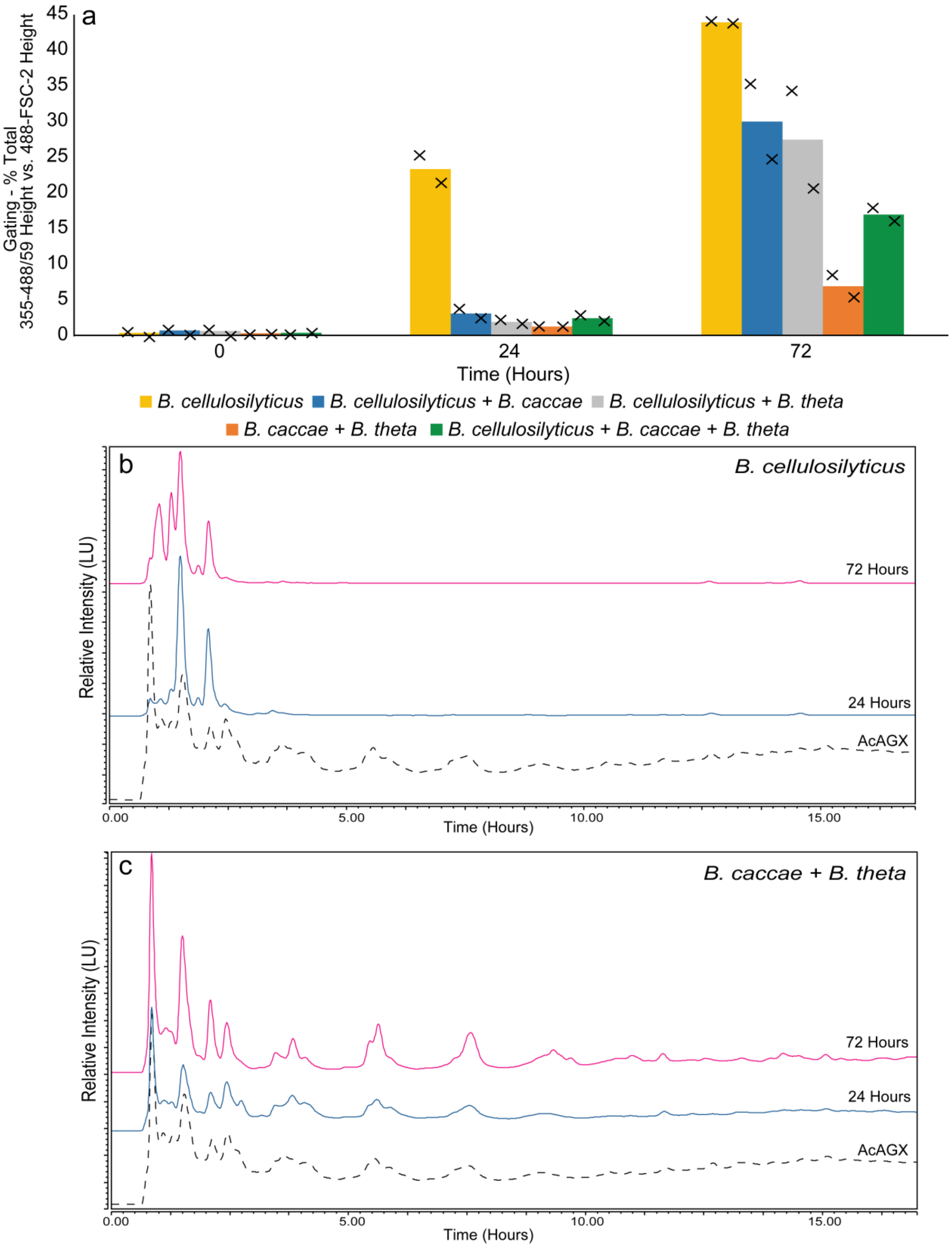

Supplementary Figure 5 – Compiled flow cytometry data and HPLC-HILIC-FLD analysis of single and co-cultured bacterial fermentations grown on 2-AB labelled AcAGX. **a** – Compiled flow cytometry data comparing a number of single and co-cultured bacterial fermentations grown

on 2-AB labelled AcAGX - supplemented with an equal amount of glucose (Data based on 2 biological replicates). **b** – HPLC-HILIC-FLD chromatograms of *B. cellulosilyticus* grown on 2-AB labelled AcAGX (supplemented with an equal amount of glucose). 2-AB labelled AcAGX (dashed line), 24 hour supernatant (blue line) and 72 hour supernatant sample (pink line). Significant degrees of degradation observed, with mainly xylose and xylobiose remaining. **c** – HPLC-HILIC-FLD chromatograms of *B. caccae* + *B. theta* grown on 2-AB labelled AcAGX (supplemented with an equal amount of glucose). 2-AB labelled AcAGX (dashed line), 24 hour supernatant (blue line) and 72 hour supernatant sample (pink line). No significant degree of degradation observed at either 24 or 72 hours.

Additional Growth Curves

As data from both Figure.9 and Supplementary Figure 5 show *B. caccae* and *B. theta* do not grow effectively on the labelled substrates used in this study. However, *B. cellulosilyticus* and *B. ovatus* both grow successfully on 2-AB labelled AcGGM and AcAGX. This trend is also observed in the modified substrates GH26-AcGGM and GH10-AGX, albeit to a lesser extent for the latter.

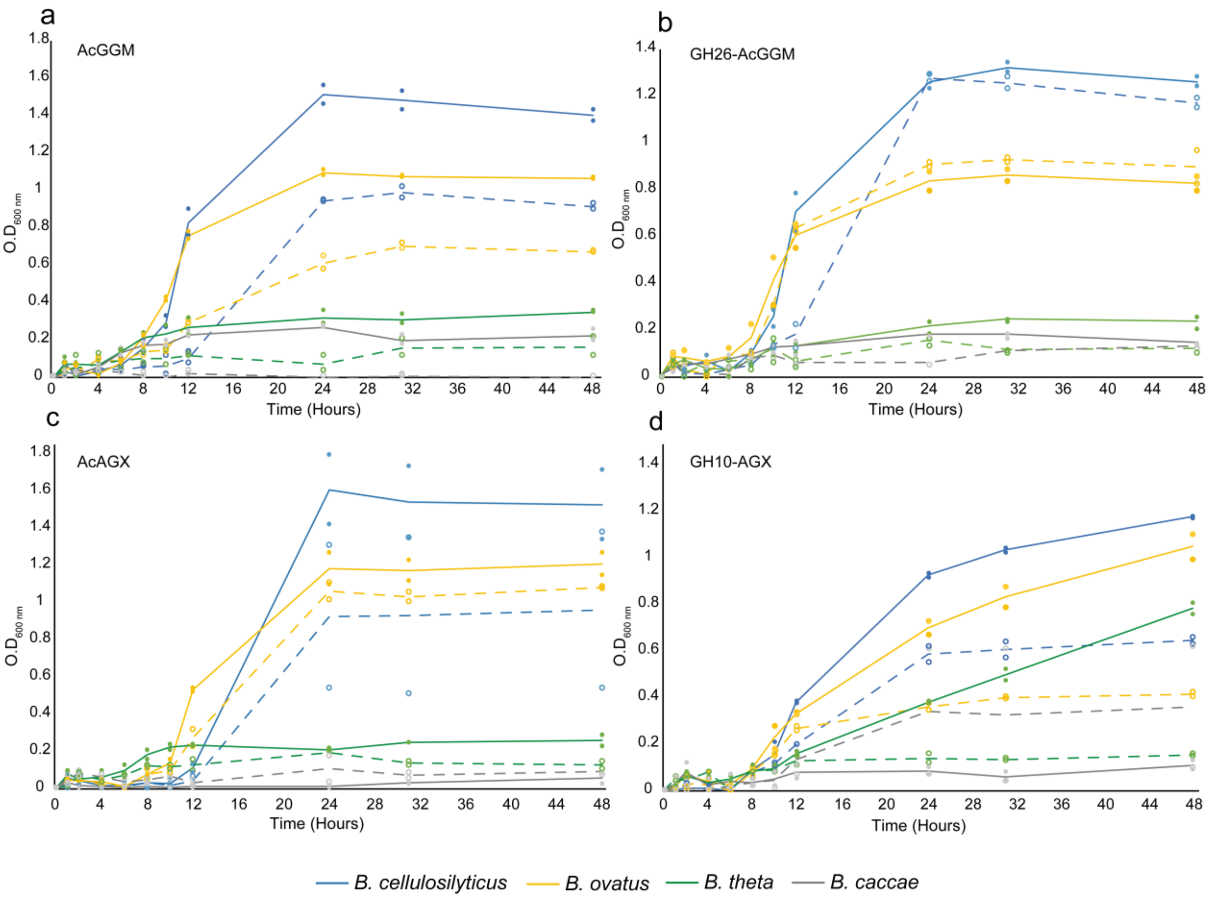

Supplementary Figure 6 – Growth curves bacteria grown on for **a** AcGGM, **b** GH26-AcGGM, **c** AcAGX, **d** GH10-AGX – Solid lines represent unlabelled substrates whilst dotted lines are the 2-AB labelled equivalents. *B. cellulosilyticus* (blue line), *B. ovatus* (yellow line), *B. theta* (green line) and *B. caccae* (grey line). All samples were precultured overnight in 5 g/L glucose and then inoculated into fresh tubes containing 5 g/L of the appropriate substrate. Data based

on 2 biological replicates (with the exception of GH26-AcGGM-2-AB for *B. caccae*, where a replicate was found to be contaminated).

SUPPLEMENTARY REFERENCES

1. Chu, A. H. A., Saati, A. E., Scarcelli, J. J., Cornell, R. J. & Porter, T. J. Reactivity-driven cleanup of 2-Aminobenzamide derivatized oligosaccharides. *Anal Biochem* **546**, 23–27 (2018).

2. Lindstad, L. J. *et al.* Human Gut Faecalibacterium prausnitzii Deploys a Highly Efficient Conserved System To Cross-Feed on β-Mannan-Derived Oligosaccharides. *MBio* e0362820 (2021) doi:10.1128/mBio.03628-20.
