# Supplementary material for: Technical pipeline for screening microbial communities as a function of substrate specificity through fluorescent labelling
Source: Commun Biol. 2022 May 11;5:444. doi: 10.1038/s42003-022-03383-z (PMC9095699; doi:10.1038/s42003-022-03383-z)
Supplement: Supplementary file 3 — Description of Additional Supplementary Files [file 42003_2022_3383_MOESM3_ESM.pdf]

Description of Additional Supplementary Files

File name: Supplementary Data 1

Description: Data for Figures 3a, 6a, 9, S5a and S6a.
